# Supplementary material for: Determining a Welfare Prioritization for Horses Using a Delphi Method
Source: Animals (Basel). 2020 Apr 9;10(4):647. doi: 10.3390/ani10040647 (PMC7222753; doi:10.3390/ani10040647)
Supplement: Supplementary file 1 [file animals-10-00647-s001.pdf]

Table 1. Percentage agreement with the ranking of horse issues from first online survey scored by experts in the second online survey. Arrows indicate whether, overall, experts considered that the issue should be moved up or down the list. Box colours indicate average agreement of 75% or higher in green, between 50% and 75% in orange, and less than 50% in red. (NB to reduce the workload of experts in the 2nd survey a small number of similar issues from the first survey were combined, hence numbers of issues are slightly fewer than in Table 2 in the Manuscript).

Rank order	***Prevalence***	***% Ranking agreement***	***Severity***	***% Ranking agreement***	***Duration***	***% Ranking agreement***
1	Lack of recognition of pain behaviour (e.g. can be misinterpreted as bolshy/naughty)	64%	Hunger	36% ↓	Obesity (e.g. due to lack of work, unrestricted grazing etc)	50%
2	Obesity (e.g. due to lack of work, unrestricted grazing etc)	36% ↓	Lack of access to clean/fresh water	57%	Lack of recognition of pain behaviour (e.g. can be misinterpreted as bolshy/naughty)	64%
3	Lack of understanding of horse welfare needs by owner/carer	39% ↑	Obesity (e.g. due to lack of work, overgrazing etc)	36% ↓	Hunger	36% ↓
4	Over-rugging (horse lacks control if too hot/cold)	43% ↑	Lack of recognition of pain behaviour (e.g. can be misinterpreted as boshly/naughty)	29% ↑	Inability to perform normal social interactions (including social isolation, stabling 24/7)	71%
5	Use of inappropriate training and handling methods	43% ↑	Delayed euthanasia	36% ↑	Lack of biosecurity and disease surveillance	57%
6	Delayed euthanasia decisions	36% ↑	Lack of biosecurity and disease surveillance	57%	Delayed euthanasia	43% ↔
7	Poorly fitting and restrictive tack	79%	Long distance transport	46% ↓	Lack of routine health care (e.g. vaccinations, dental, parasite prevention)	79%
8	Poor owner knowledge of horse care	43% ↑	Inability to perform normal social interactions (including social isolation, stabling 24/7)	43% ↑	Large worm burdens	64%
9	Unstable social groups	57%	Overworking	57%	Poor owner knowledge of horse care	57%
10	Lack of biosecurity and disease surveillance	50%	Poorly fitting and restrictive tack	79%	Poor pasture management	50%

11	Inability to perform normal social interactions (e.g. social isolation, stabling 24/7)	57%	Abandonment or stray horses	86%	Over-rugging (horse lacks control if too hot/cold)	43% ↓
12	No regulation of establishments or service providers	54%	Unsuitable diets for equine feeding behaviour (e.g. feeding methods of stabled horses, turned out 24/7)	50%	Unsuitable diets for equine feeding behaviour (e.g. feeding methods of stabled horses, turned out 24/7)	57%
13	Unsuitable diets for equine feeding behaviour (e.g. feeding methods of stabled horses, turned out 24/7)	57%	Overweight riders for horse	64%	Use of food supplements	29% ↓
14	Negative affective states (fear, frustration, boredom, depression, anxiety)	57%	Use of inappropriate training and handling methods	43% ↑	No regulation of establishments or service providers	46% ↓
15	Poor pasture management	57%	Unavoidable aggressive social interactions (e.g. restricted resources in groups, etc)	57%	Negative affective states (fear, frustration, boredom, depression, anxiety)	36% ↑
16	Neglect	86%	Neglect	57%	Lack of understanding of horse welfare needs	36% ↑
17	Horse passports not fit for purpose	54%	Large worm burdens	64%	Overweight riders for horse	71%
18	Overworking	36% ↓	Hiring horses to unsuitable riders	86%	Poorly fitting and restrictive tack	64%
19	Poor health knowledge by owner	64%	Poor weaning methods	50%	Use of inappropriate training and handling methods	50%
20	Poor weaning methods	64%	Poor health knowledge by owner	71%	Neglect	64%
21	Overweight riders for horse	64%	No regulation of establishments or service providers	54%	Poor weaning methods	46% ↑
22	Use of food supplements	36% ↓	Over-rugging (horse lacks control if too hot/cold)	50%	Indiscriminate breeding (including of compromised animals)	71%
23	Indiscriminate breeding (including of compromised animals)	64%	Poor owner knowledge of horse care	62%	Unstable social groups	64%
24	Being ridden	21% ↓	Horse passports not fit for purpose	46% ↓	Abandonment or stray horses	79%
25	Fear, stress or injury from use in work sport or entertainment	43% ↔	Negative affective states (fear, frustration, boredom, depression, anxiety)	43% ↑	Poor owner health knowledge	69%
26	Lack of routine health care (e.g. vaccinations, dental, parasite prevention)	71%	Unstable social groups	29% ↑	Lack of access to clean/fresh water	57%
27	Hiring horses to unsuitable riders	79%	Poor pasture management	64%	Hiring horses to unsuitable riders	71%

28	Poor transport of horses on domestic journeys	64%	Lack of understanding of horse welfare needs	21% ↑	Unavoidable aggressive social interactions (e.g. restricted resources in groups, etc)	79%
29	Long distance transport	71%	Fear, stress or injury from use in work sport or entertainment	43% ↑	Long distance transport	71%
30	Unavoidable aggressive social interactions (e.g. restricted resources in groups, etc)	71%	Indiscriminate breeding (including of compromised animals)	43% ↑	Fear, stress or injury from use in work sport or entertainment	57%
31			Lack of routine health care (e.g. vaccinations, dental, parasite prevention)	57%	Flygrazing	86%
32			Poor transport of horses on domestic journeys	93%	Overworking	86%
33			Lack of easy access to health care	79%	Lack of easy access to health care	79%
